# Self-Reported Cognitive Impairment Across Racial/Ethnic Groups in the United States, National Health Interview Survey, 1997–2015

**DOI:** 10.5888/pcd15.170338

**Published:** 2018-01-11

**Authors:** Huabin Luo, Gary Yu, Bei Wu

**Affiliations:** 1 Department of Public Health, East Carolina University, Greenville, North Carolina; 2 Rory Meyers College of Nursing, New York University, New York, New York; 3 NYU Aging Incubator, New York, New York

## Abstract

**Introduction:**

The primary objectives of this study were 1) to examine trends of self-reported cognitive impairment among 5 major racial/ethnic groups during 1997–2015 in the United States and 2) to examine differences in the trends across these groups.

**Methods:**

Data were from the National Health Interview Survey (NHIS). The sample consisted of 155,682 people aged 60 or older. Respondents were asked to report whether any family member was “limited in any way because of difficulty remembering or because of experiencing periods of confusion.” Race/ethnicity categories were non-Hispanic white, non-Hispanic black, Native American, Hispanic, and Asian. We applied hierarchical age–period–cohort cross-classified random-effects models for the trend analysis. All analyses accounted for the complex survey design of NHIS.

**Results:**

The overall rate of self-reported cognitive impairment increased from 5.7% in 1997 to 6.7% in 2015 (*P* for trend <.001). Among non-Hispanic white respondents, the rate increased from 5.2% in 1997 to 6.1% in 2015 (slope = 0.14, *P* for trend <.001). We observed no significant trend in rate of cognitive impairment in other groups. After we controlled for covariates, we found that Asian (B = 0.31), non-Hispanic black (B = 0.37), Hispanic (B = 0.25), and Native American (B = 0.87) respondents were more likely than non-Hispanic white respondents to report cognitive impairment (*P* <.001 for all).

**Conclusion:**

We found an increased rate of self-reported cognitive impairment in older adults of 5 major racial/ethnic groups from 1997 through 2015 in the United States. However, the rate of self-reported cognitive impairment was low, which may suggest underreporting. There is a need to further promote awareness of the disease among individuals, family members, and health care providers.

## Introduction

The aging population is increasing rapidly in the United States. The size of the US population aged 65 or older is projected to be 88.5 million in 2050, more than double the size of this population (40.2 million) in 2010 (1). The population of older adults will also become more racially and ethnically diverse. By 2050, the proportion of white older adults will account for 77% of the population aged 65 or older, down from 87% in 2010, whereas the proportion for black older adults will be 12% (9% in 2010), Asian older adults 9% (3% in 2010), and Hispanic older adults 20% (7% in 2010) (1).

With the rapid increase in the population of older adults, the size of the population with cognitive impairment and dementia will expand (2). Approximately 5.5 million Americans had Alzheimer’s disease in 2017. This number is expected to grow to 13.8 million by 2050 (3). The age-adjusted death rate of Alzheimer’s disease in the United States increased by 54.5% from 1999 to 2014, from 16.5 per 100,000 population to 25.4 per 100,000 population. Significant increases occurred in all age groups, both sexes, and all racial/ethnic groups (4).

Cognitive impairment may be a precursor to dementia (5). Early detection of cognitive impairment would facilitate timely medical treatments, appropriate care planning, and prevention efforts, which would ultimately reduce health care costs. Data on self-reported cognitive impairment are limited (6–8). Most previous studies have evaluated cognitive impairment by using screening tests or clinical examination (9–11). Some studies reported racial/ethnic disparities in cognitive impairment in the United States (12–14). Older African American adults are about twice as likely and older Hispanic adults are about 1.5 times as likely to have Alzheimer’s disease or other dementias as older non-Hispanic white adults (15). An important limitation of previous research on cognitive functioning is that it focused mostly on 3 racial/ethnic groups (white, black, and Hispanic) or fewer (14). No studies have examined the rate and trend of cognitive impairment in Asian or Native American adults in the United States. Information on the cognitive health for subpopulations is needed for health planning and delivery of culturally competent services. Hence, new research is warranted to assess the rate of cognitive impairment in various racial/ethnic groups in the United States.

This study aimed to fill the gap in the literature by using data from a nationally representative population-based survey, the National Health Interview Survey (NHIS) (16). The objectives of the study were 1) to examine the trends in rate of self-reported cognitive impairment among American adults aged 60 or older in 5 major racial/ethnic groups from 1997 through 2015 and 2) to examine differences in trends of self-reported cognitive impairment across these racial/ethnic groups.

## Methods

We collected data on the following racial/ethnic groups: non-Hispanic white, non-Hispanic black, Hispanic, Native American, and Asian. Data were from the NHIS, a cross-sectional household interview survey conducted annually by the National Center for Health Statistics, which is part of the Centers for Disease Control and Prevention (CDC). NHIS obtains information through in-person interviews with household respondents to monitor the health of the US population. The multistage area probability design provides a representative sample of the civilian noninstitutionalized population residing in the United States at the time of the interviews. The analytical sample for this study included 155,682 adults aged 60 or older in the NHIS adult samples from 1997 through 2015.

The outcome variable was self-reported cognitive impairment. In the NHIS family health status and limitation questionnaire, respondents are asked whether anyone in the household has a limitation in his or her everyday activities (eg, activities of daily living, instrumental activities of daily living, play, school, and work; difficulties in walking or in remembering) as a result of a physical, mental, or emotional health problem. The responses may be self-reported or reported by an adult household member or by proxy for other household members not present at the interview. We classified a person as having cognitive impairment if the answer was yes to the question on “limited in any way because of difficulty remembering or because of experiencing periods of confusion.” This measure provides reliable estimates of cognitive status (17). (Another related NHIS question asks about cognitive impairment due to senility; that is, a limitation due to senility, Alzheimer’s disease, or another aging-related cognitive impairment. We did not use this measure in our analysis because of the small sample sizes, particularly for racial/ethnic minority groups.)

We used 3 sociodemographic variables as covariates: age (60–64 y, 65–69 y, 70–74 y, 75–79 y, 80–84 y, and ≥85 y), sex (male or female), and marital status (married or living with partner vs otherwise). We also used 2 variables of socioeconomic status as covariates: education level (some college or more vs others) and family income level. Family income was defined as the ratio of total family income to the federal poverty level (FPL), which was calculated as the family’s income in the most recent calendar year divided by the applicable poverty threshold based on the size of the family. We classified this poverty ratio variable into 4 categories: less than 150% of the FPL, 150% to 249% of the FPL, 250% to 499% of the FPL, and 500% or more of the FPL.

In addition, we used 2 health-related variables as covariates. We collected data on whether or not (yes or no) respondents self-reported the following 5 diseases: heart attack, diabetes, high blood pressure, coronary heart disease, and stroke. We also collected data on body mass index (BMI), defined as weight in kilograms divided by height in meters squared (kg/m^2^), and we used the following BMI categories (18): underweight (<18.5 kg/m^2^), normal weight (18.5–24.9 kg/m^2^), overweight (25.0–29.9 kg/m^2^), and obese (≥30.0 kg/m^2^).

Period and birth cohort were also included as covariates. The period was the survey year — the 19 waves of NHIS surveys from 1997 through 2015. We created 9 birth cohorts with 5- or 6-year intervals from 1912–1917 to 1953–1957. 

We first calculated the rate of cognitive impairment in the various age groups, survey periods, and birth cohorts in the 5 racial/ethnic groups and assessed the significance of the slopes of trend lines (19). Because of the small sample size of Native American adults in each wave of NHIS, we calculated 3-year moving average rates of cognitive impairment (2-year at both end points: 1997–1998 for the first period, 2014–2015 for the last period, and 3-year for the middle periods), and we used the average of the survey weight variable according to 2 or 3 years included. In addition, we compared the slopes of trend lines of period (survey year) to assess the difference in the rate of change over time by using *t* tests.

Second, because NHIS data are cross-sectional, to control for age, survey period, and birth cohort effects, we applied the hierarchical age–period–cohort (HAPC) cross-classified random-effects model (CCREM) (20) to examine the trends of cognitive impairment. Data analyses were conducted by using SAS PROC GLIMMIX (21) with DIST=binary. The HAPC–CCREM model can effectively estimate any random clustering effects at higher-level cross-classified units such as survey periods and birth cohorts (20). Predicted probability of self-reported cognitive impairment (controlling for all covariates) was estimated for the 5 racial/ethnic groups by age group and birth cohort. We used sampling weights in analyses. A more conservative level of significance was set at .01, instead of the standard .05 level, to account for multiple pairwise comparisons to a reference category using a Bonferroni correction (0.05/5 = 0.01) (22).

In the HAPC–CCREM model, age effects refer to variation associated with different age groups, reflect biological and social processes of aging internally to individuals, and represent developmental changes during the life course. Period effects refer to changes in social, economic, technological, or physical environments affecting all age groups simultaneously at the time health is measured. Finally, cohort effects refer to variation among persons in different birth cohorts. A person in a given birth cohort experiences the same historical and social events at various stages of his or her life course as other people in that birth cohort. Thus, not taking into account cohort effects would possibly lead to biased estimate of trends of social inequalities in the outcome of interest (20).

## Results

Among the 5 racial/ethnic groups, the non-Hispanic white group was the oldest, and the Hispanic group the youngest. Asian respondents had the largest proportion of adults who had some college or more (62.1%) and were married or living with a partner (70.4%) (Table 1). Hispanic respondents had the lowest family income: 65.6% were in the first or second income categories. The rates of high blood pressure (71.5%) and obesity (38.4%) were highest among non-Hispanic black respondents. Native American respondents had the highest rates of heart attack (14.2%), diabetes (31.4%), coronary heart disease (16.6%), and stroke (10.9%).

**Table 1:** Sample Characteristics, by Race/Ethnicity, National Health Interview Survey, 1997–2015

Variable	Non-Hispanic White, % (n = 116,445)	Non-Hispanic Black, % (n = 19,975)	Native American, % (n = 796)	Hispanic, % (n = 15,553)	Asian, % (n = 2,913)	*P* Value^a^
**Age, y**						
60–64	28.4	32.5	35.0	32.8	32.9	<.001
65–69	22.5	24.0	22.8	25.6	25.3	
70–74	18.5	18.7	19.7	18.6	18.3	
75–79	12.6	11.0	9.4	11.2	10.4	
80–84	9.3	7.3	7.9	6.7	6.4	
≥85	8.7	6.6	5.1	5.3	6.7	
**Female**	55.0	58.9	51.7	55.7	51.8	<.001
**Married or living with partner**	63.1	42.1	51.0	58.3	70.4	<.001
**Some college or more**	48.5	34.3	39.2	24.9	62.1	<.001
**Ratio of family income to federal poverty level**						
<150%	17.4	41.5	38.6	42.3	22.9	<.001
150%–249%	22.0	21.9	23.5	23.3	16.0	
250%–499%	33.6	24.1	22.8	23.3	28.8	
≥500%	27.1	12.6	15.1	11.1	32.3	
**Chronic disease**						
Heart attack	10.2	8.4	14.2	7.5	6.4	<.001
Diabetes	15.7	28.3	31.4	27.3	20.6	<.001
High blood pressure	53.8	71.5	62.9	55.2	55.9	<.001
Coronary heart disease	13.4	11.0	16.6	10.3	10.3	<.001
Stroke	7.1	9.9	10.9	7.0	6.0	<.001
**Body mass index, kg/m^2^**						
<18.5	2.0	1.5	2.8	1.1	3.4	<.001
Normal (18.5–24.9)	33.8	24.8	26.8	26.4	53.9	
Overweight (25.0–29.9)	36.8	35.3	33.4	40.5	30.5	
Obese (≥30.0)	27.5	38.4	37.0	32.0	12.1	

a According to χ^2^ tests.

The overall rate of self-reported cognitive impairment increased from 5.7% (95% confidence interval, 5.2%–6.3%) in 1997 to 6.7% (6.1%–7.3%) (*P* for trend <.001). The rate increased from 5.2% in 1997 to 6.1% in 2015 among non-Hispanic white respondents (*P* for trend < .001). We found no significant trends in cognitive impairment among non-Hispanic black, Native American, Hispanic, or Asian respondents (Table 2).

**Table 2:** Weighted Prevalence of Cognitive Impairment, by Racial/Ethnic Group, National Health Interview Survey,1997–2015

Year	Percentage					
	Non-Hispanic White	Non-Hispanic Black	Native American^a^	Hispanic	Asian	Overall
1997	5.2	9.1	NA	9.3	NA	5.7
1998	5.0	8.0	6.5	6.2	7.4	5.4
1999	4.3	7.5	9.1	6.5	3.7	4.7
2000	5.0	10.2	12.7	7.3	9.4	5.6
2001	5.6	11.1	20.6	8.9	6.1	6.3
2002	6.0	8.6	20.6	7.5	14.7	6.5
2003	6.0	11.4	13.7	7.0	7.3	6.6
2004	5.9	8.3	7.6	8.1	7.6	6.3
2005	6.2	9.9	9.6	8.3	7.8	6.7
2006	5.7	9.8	13.5	7.5	6.2	6.2
2007	6.2	12.3	14.1	8.1	8.1	6.9
2008	5.3	9.1	10.3	6.1	7.0	5.8
2009	6.1	11.4	10.1	6.6	4.4	6.6
2010	6.0	8.4	11.4	8.4	5.6	6.4
2011	6.5	10.2	13.3	8.7	10.7	7.2
2012	5.8	9.5	15.3	9.3	6.1	6.5
2013	6.3	10.8	16.0	8.0	6.1	6.9
2014	6.2	9.7	16.9	8.9	5.0	6.8
2015	6.1	9.2	16.2	8.7	5.3	6.7
*P* value for trend	<.001	.30	.27	.15	.28	<.001

a 3-year average.

We found no significant differences in the rate of change in slopes between non-Hispanic white and non-Hispanic black respondents (*t* = 0.24, *P* = .81), non-Hispanic white and Hispanic respondents (*t* = 0.18, *P* = .86), or non-Hispanic white and Asian respondents (*t* = 0.17, *P* = .10). We did not compare the slope of non-Hispanic white respondents and Native American respondents because rates for Native American respondents were calculated in 3-year moving averages.

**Figure 1:**
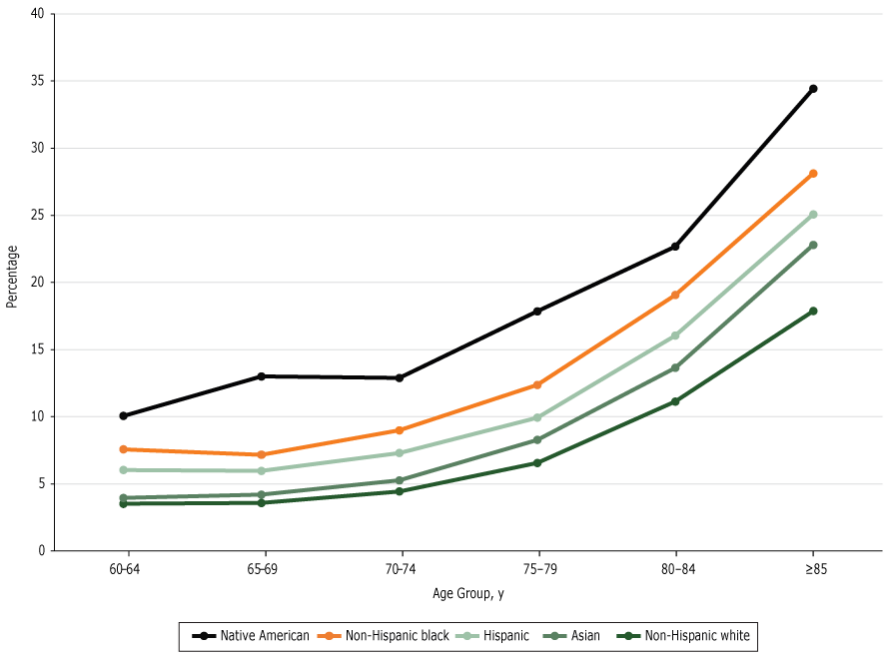
Predicted probability of reporting cognitive impairment, by age group. Data are from the National Health Interview Survey, 1997–2015. **Table:** 

**Figure 2:**
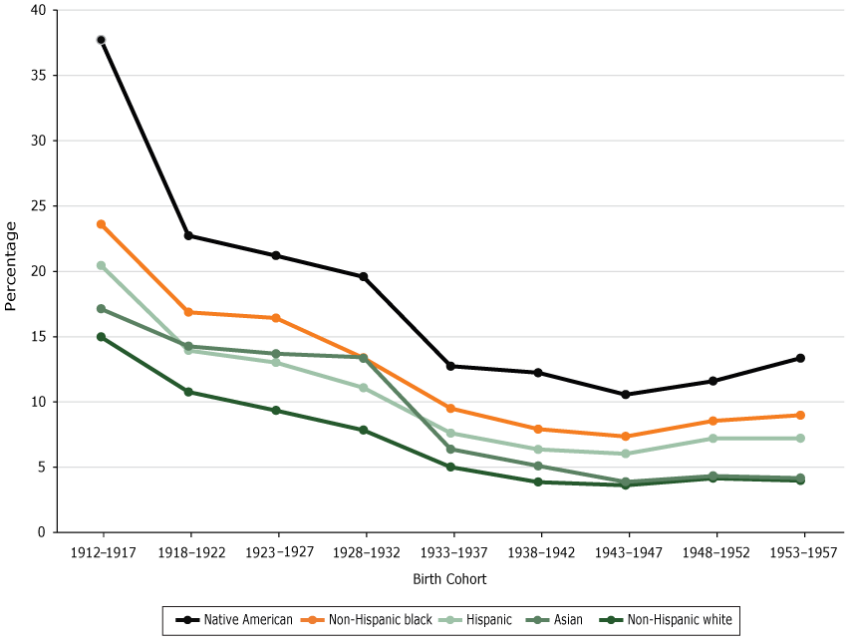
Predicted probability of reporting cognitive impairment, by birth cohort. Data are from the National Health Interview Survey, 1997–2015. **Table:** 

Overall, the rate of cognitive impairment increased with age in all 5 racial/ethnic groups (*P* < .01 for all groups), with a sharper increase in those 85 years or older (Figure 1). Across the 9 birth cohorts, the rate was lowest among non-Hispanic white respondents until the 1943–1947 birth cohort, when the rate among Asian respondents was similar to the rate among non-Hispanic white respondents (Figure 2).

Age Group, y	Non-Hispanic White, %	Non-Hispanic Black, %	Native American, %	Hispanic, %	Asian American, %
60–64	3.5	7.6	10.1	6.0	4.0
65–69	3.6	7.2	13.0	6.0	4.2
70–74	4.4	9.0	12.9	7.3	5.3
75–79	6.6	12.4	17.9	9.9	8.3
80–84	11.1	19.1	22.7	16.0	13.6
≥85	17.9	28.1	34.4	25.1	22.8

Birth Cohort	Non-Hispanic White	Non-Hispanic Black	Native American	Hispanic	Asian
1912–1917	15.0	23.6	37.7	20.4	17.1
1918–1922	10.8	16.9	22.7	13.9	14.3
1923–1927	9.3	16.4	21.2	13.0	13.7
1928–1932	7.8	13.4	19.6	11.1	13.4
1933–1937	5.0	9.5	12.7	7.6	6.4
1938–1942	3.9	7.9	12.2	6.4	5.1
1943–1947	3.6	7.4	10.6	6.0	3.9
1948–1952	4.1	8.5	11.6	7.2	4.3
1953–1957	4.0	9.0	13.3	7.2	4.2

In the HAPC–CCREM model, compared with non-Hispanic white respondents, non-Hispanic black respondents (B = 0.37, *P* < .001), Native American respondents (B = 0.87, *P* < .001), Hispanic respondents (B = 0.25, *P* < .001), and Asian respondents (B = 0.31, *P* < .001) were more likely to report cognitive impairment (Table 3). Parameter estimates on all other covariates were significant (*P* < .001): lower education level; lower family income level; having a heart attack, diabetes, high blood pressure, coronary heart disease, or stroke; and underweight were all risk factors of cognitive impairment. The interaction term race/ethnicity by some college or more was significant for all groups, indicating that college education reduced the odds of having cognitive impairment, except for the Hispanic group.

**Table 3:** Hierarchical Age–Period–Cohort Cross-Classified Model Results of Factors Associated With Self-Reported Cognitive Impairment, National Health Interview Survey,1997–2015

Variable	B (Standard Error)	*P* Value
**Fixed effects**		
Intercept	−2.15 (0.05)	<.001
Age (centered)	0.04 (0)	<.001
Age^2^	0 (0)	<.001
**Sex**		
Male	Reference	
Female	−0.07 (0)	<.001
**Marital status**		
Not married or living with partner	Reference	
Married or living with partner	−0.33 (0)	<.001
**Race/ethnicity**		
Non-Hispanic white	Reference	
Non-Hispanic black	0.37 (0)	<.001
Native American	0.87 (0.01)	<.001
Hispanic	0.25 (0)	<.001
Asian	0.31 (0.01)	<.001
**Education**		
<Some college or more	Reference	
Some college or more	−0.20 (0)	<.001
**Ratio of family income to federal poverty level**		
<150%	Reference	
150%–249%	−0.33 (0)	<.001
250%–499%	−0.43 (0)	<.001
≥500%	−0.83 (0)	<.001
**Body mass index, kg/m^2^**		
Underweight (<18.5)	Reference	
Normal (BMI 18.5–24.9)	−0.65 (0)	<.001
Overweight (BMI 25.0–29.9)	−0.93 (0)	<.001
Obese (≥30.0)	−0.74 (0)	<.001
**Chronic disease^a^**		
Heart attack	0.16 (0)	<.001
Diabetes	0.47 (0)	<.001
High blood pressure	0.09 (0)	<.001
Coronary heart disease	0.32 (0)	<.001
Stroke	1.44 (0)	<.001
**Race/ethnicity × college or more**		
Non-Hispanic white × college or more	Reference	
Non-Hispanic black × college or more	−0.06 (0.01)	<.001
Native American × college or more	−0.20 (0.02)	<.001
Hispanic × college or more	−0.01 (0.01)	.13
Asian × college or more	−0.27 (0.01)	<.001
**Random effects **		
Survey period	0.01 (0)	.001
1997	−0.10 (0.03)	.001
1998	−0.17 (0.03)	<.001
1999	−0.26 (0.03)	<.001
2000	−0.14 (0.03)	<.001
2001	0.03 (0.03)	.35
2002	0.03 (0.03)	.32
2003	0.05 (0.03)	.04
2004	−0.03 (0.03)	.21
2005	0.12 (0.03)	<.001
2006	−0.01 (0.03)	.58
2007	0.15 (0.03)	<.001
2008	−0.18 (0.03)	<.001
2009	0.04 (0.03)	.12
2010	0.07 (0.03)	.02
2011	0.13 (0.03)	<.001
2012	0.05 (0.03)	.08
2013	0.09 (0.03)	.001
2014	0.13 (0.03)	<.001
2015	0.00 (0.03)	.98
**Cohort**	0.01 (0.01)	.02
1912–1917	0.01 (0.04)	.78
1918–1922	−0.15 (0.04)	<.001
1923–1927	−0.06 (0.04)	.14
1928–1932	−0.07 (0.04)	.06
1933–1937	0.00 (0.04)	.93
1938–1942	−0.07 (0.04)	.07
1943–1947	−0.03 (0.04)	.45
1948–1952	0.19 (0.04)	<.001
1953–1957	0.18 (0.04)	<.001

a Reference group for each category is absence of the disease.

Overall, the survey period had a significant random effect (B = 0.01, *P* = .001). The birth cohort 1918–1922 had negative effects, whereas the birth cohorts 1948–1952 and 1953–1957 had positive effects. However, the overall random cohort effects were not significant (Table 3).

## Discussion

To our knowledge, our study is the first to provide a national trend estimate on self-reported cognitive impairment among non-Hispanic white, non-Hispanic black, Hispanic, Native American, and Asian adults aged 60 or older in the United States from 1997 to 2015. Our findings indicate a significant increasing trend in self-reported cognitive impairment. Racial/ethnic differences in self-reported cognitive impairment persisted throughout the study period; the rate of self-reported cognitive impairment was lowest among non-Hispanic white adults.

A study in 2014 analyzed the same NHIS data and found that cognitive impairment in adults aged 18 or older increased from 2.0% in 1998 to 3.3% in 2011 (8), but cognitive impairment was not a focus, and the study covered a shorter period (8).

It is interesting that our data showed an increasing trend in self-reported cognitive impairment in adults aged 60 or older. Other recent studies that used data from cognitive tests and clinical assessments found a declining trend in dementia in the United States (9,23). A report in 2016 on the Framingham Heart Study showed that the temporal trends of incidence of dementia among participants aged 60 or older declined by about 20% per decade between 1977 and 2008: the 5-year age- and sex-adjusted dementia rates were 3.6% during 1977–1983, 2.8% during 1986–1991, 2.2% during 1992–1998, and 2.0% during 2004–2008 (10). In addition, an analysis of Health and Retirement Study (HRS) data found that the prevalence of cognitive impairment with no dementia in adults aged 65 or older decreased significantly from 21.2% in 2000 to 18.8% in 2012 (9). The HRS assesses cognitive function by using a multidimensional measure based on a modified version of the Telephone Interview Cognitive Screen and tests of immediate and delayed verbal recalls (9).

Nevertheless, it is not appropriate to make direct comparisons among these studies because of differences in definitions, operationalization of cognitive impairment, and study designs. The significant increasing trend of self-reported cognitive impairment found in our study might suggest that awareness of cognitive impairment has improved in the United States, especially in recent years (ie, after 2010). The heightened public attention to and interest in Alzheimer’s disease might have contributed to the increased awareness. Indeed, efforts to improve awareness and perception of cognitive health has been ongoing, including CDC’s and the Alzheimer’s Association’s Health Brain Initiative, the Alzheimer’s Association’s Maintain Your Brain workshops and community-based demonstration projects to promote cognitive health, and initiatives by the American Association of Retired Persons (24).

Several factors should be considered when interpreting our findings on the increasing trend of cognitive impairment. First, regardless of the increasing trend, an overall rate, ranging from 4.7% to 6.7% during the study period, may be underreported. When we analyzed data on a subset of our sample (those aged 65 or older), the rate of self-reported cognitive impairment was 6.3% in 2000 and 7.5% in 2012. These rates are still lower than the rates shown by HRS data for the same age group (21.2% in 2000 and 18.8% in 2012) (9).

Second, we found an increasing trend in cognitive impairment only among non-Hispanic white adults. Overall, racial/ethnic minority groups in our study had higher rates of self-reported cognitive impairment than did the non-Hispanic white group. Public education is needed to promote awareness of this disease, especially among racial/ethnic minority groups, who may have different cultural beliefs and perceptions of disease and aging than non-Hispanic white groups (25). Members of racial/ethnic minority groups may be less likely to seek treatment for psychiatric symptoms because of lack of access to care and/or cultural stigmatization (26). Moreover, racial/ethnic minority populations may be less likely than the non-Hispanic white population to accept treatment for depression (27). If the symptoms of depression are left untreated, cognitive health and quality of life may decline.

Third, the HAPC model results showed racial/ethnic disparities: racial/ethnic minority adults were more likely than non-Hispanic white adults to report cognitive impairment. These findings are consistent with previous findings indicating that white adults were less likely to have cognitive impairment than black or Hispanic adults (12,13). A recent study that used HRS data found that Hispanic and black adults had lower levels of cognitive function than white adults: in a cognitive test having a maximum score of 27 points, Hispanic adults scored 13.7, black adults scored 13.3, and white adults scored 16.3 (12). In addition, consistent with prior research, our study showed that diseases such as diabetes and stroke were associated with higher odds of self-reported cognitive impairment (28). The persistent racial/ethnic disparities in chronic disease risk factors may partially account for the higher rate of cognitive impairment among racial/ethnic minority groups. National Health and Nutrition Examination Survey data for 1988–1994 to 1999–2004 showed that racial/ethnic disparities in cardiovascular disease risk factors between non-Hispanic whites and non-Hispanic blacks and between non-Hispanic whites and Mexican Americans did not improve; for some health outcomes, such as obesity and hypercholesterolemia, the gap even widened (29).

No overall birth cohort effects were observed in our study, although the cohort 1918–1922 was less likely than other cohorts to self-report cognitive impairment, whereas the 2 youngest cohorts (ie, 1948–1952 and 1953–1957) were more likely than other cohorts to self-report cognitive impairment. Finally, we found a significant interaction effect between education and race (except in Hispanics), indicating a similar effect of education on cognitive impairment across groups. Education levels have increased in the United States in the past several decades (30), and education is shown to protect against cognitive decline (31). A higher level of education may contribute to the ability to recognize cognitive decline. Education could explain why younger birth cohorts were more likely than older cohorts in our study to report cognitive impairment. Thus, our study indicates that the role of education in self-reported cognitive impairment is twofold: a higher level of education protects against cognitive decline and contributes to greater self-awareness of cognitive impairment.

The data used in our study have several limitations. First, the data were self-reported and thus are subject to reporting bias. Respondents may intentionally withhold information because of stigma, or they may not have been able to provide a reliable answer because they were cognitively impaired. Self-reported cases of cognitive impairment cannot be validated through clinical assessment. Also, proxy-reported data could introduce bias. Proxies might misreport the information for various reasons, including lack of knowledge and stigma. However, studies showed that proxy-reported data on cognition status of family members may be more reliable than self-reported data (32). Second, the sample size for the Native American group was small for calculating yearly estimates. Future surveys should increase the sample size for the Native American group because Native Americans are at higher risk of many chronic diseases that are associated with cognitive impairment, including cardiovascular disease and diabetes (14). Third, our study did not include residents in long-term care facilities, who are more likely to be cognitively impaired than community-dwelling older adults.

From 1997 to 2015, NHIS data showed that the overall rate of self-reported cognitive impairment increased among the 5 major racial/ethnic groups in the United States. However, by racial/ethnic group, the increasing trend was significant only among the non-Hispanic white group. Significant racial/ethnic disparities persisted during the study period. Our findings may reflect an increased self-awareness of cognitive impairment in the United States. They also underscore the need to further promote awareness of cognitive impairment, especially among racial/ethnic minority populations. Health education is needed for individuals, family members, and health care providers to improve awareness and knowledge of signs and early symptoms of Alzheimer’s disease and other forms of dementia.
